# Knowledge and acceptance associated with medication adherence among hypertension individuals in Aceh province, Indonesia

**DOI:** 10.1016/j.heliyon.2024.e29303

**Published:** 2024-04-05

**Authors:** Sri Andala, Hizir Sofyan, Kartini Hasballah, Marthoenis

**Affiliations:** a Graduate School of Mathematics and Applied Sciences, Universitas Syiah Kuala, Banda Aceh, 23111, Indonesia; b STIKes Muhammadiyah Lhokseumawe, Lhokseumawe, 24300, Indonesia; c Dinas Kesehatan Kota Lhokseumawe, Lhokseumawe, 24300, Indonesia; d Department of Statistics, Faculty of Math and Science, Universitas Syiah Kuala, Banda Aceh, 23111, Indonesia; e Department of Pharmacology, Faculty of Medicine, Universitas Syiah Kuala, Banda Aceh, 23111, Indonesia; f Department of Psychiatry and Mental Health Nursing, Universitas Syiah Kuala, Banda Aceh, 23111, Indonesia

**Keywords:** Hypertension, Knowledge, Acceptance, Indonesia, Medication adherence

## Abstract

Low adherence to anti-hypertensive medication is observed among individuals in Aceh, the westernmost province of Indonesia. Since uncontrolled hypertension has the potential to develop into a life-threatening disease, exploring medication adherence among this specific population is essential. Therefore, this study aimed to evaluate knowledge and acceptance associated with medication adherence among hypertensive individuals in Aceh Province. A cross-sectional study was conducted from March to July 2023 on 534 respondents diagnosed with hypertension, who were selected using the random sampling method. Demographic characteristics collected included body height and weight, age, gender, education, ethnicity, and occupation. Acceptance and knowledge were measured through a set of standardized questionnaires while the Morisky Medication Adherence Scale-8 was used for evaluating medication adherence. Logistic regression with a multinomial model was used to assess the correlations of acceptance and knowledge with medication adherence. The results showed that only 28.5 % of the respondents had high adherence to anti-hypertensive medication. Furthermore, a high level of acceptance towards hypertension significantly predicted medication adherence (*p* < 0.001; OR = 9.14 [95%CI: 3.49–23.94]). Knowledge about dosing frequency, the benefits of low-fat and sodium diets, and the negative impacts of drinking alcohol were correlated with high-level adherence (*p* < 0.01). Meanwhile, knowledge about renal complications correlated negatively with adherence level (*p* = 0.002; OR = 0.32 [95%CI: 0.16–0.66]). In conclusion, this study showed that acceptance and knowledge of hypertension correlated with the level of medication adherence.

## 1 Introduction

Hypertension is a leading cause of global cardiovascular diseases and premature death, prompting focused attention on treatment from various studies and public health practitioners [1]. This disease significantly contributed to disability-adjusted life years (DALYs), which increased from 2.3 million to 2.5 million between 2017 and 2018 in the United States [2]. Regarding low- and middle-income countries, the prevalence of hypertension reached 31.5 %, due to low awareness and uncontrolled blood pressure [3]. Based on the basic health research (RIKESDAS) 2018, the prevalence among the adult population was 34.1 % in Indonesia, showing an 8.3 % increase from the 2013 survey [4]. The number is predicted to increase due to several factors such as diabetes, obesity, smoking, sedentary lifestyle, and stroke prevalence [5]. At the provincial level, the prevalence ranges from 44.13 % in Kalimantan Selatan to 22.22 % in Papua [6].

Aceh is a province located in the westernmost island of Indonesia, with a 26.5 % prevalence of hypertension [4]. Despite the lower prevalence compared to the national average, blood pressure control by pharmacological treatment remains a major challenge [7,8]. As the only Indonesian province to implement Islamic Law (Syariah Law), Aceh has unique demographic characteristics. In this province, spirituality has become a significant factor in managing diseases, including hypertension [9]. Individuals with hypertension can develop serious complications such as cardiovascular and renal diseases, often exacerbated by poor adherence to medication, which is strongly correlated with inadequate blood pressure control [10]. In the real-world setting, Acehnese population commonly deviates from maintaining medication regimen after the perception of healthy status. A previous regency-specified study reported that the prevalence of adherence to medication in Aceh ranged between 33.3 % and 64.3 % [11]. However, certain limitations such as a low number of samples (≤56 participants) and poor protocol contribute to the minimal level of evidence [11]. This shows the need to investigate the factors affecting adherence to medication among individuals with hypertension in this particular population.

Acceptance of the chronic disease could affect medication adherence, although the relationship is relatively complex [[12], [13], [14]]. The level of acceptance towards specific diseases reflects individuals' capability to acknowledge the condition, adapt emotionally, and effectively cope with the challenges. Knowledge about hypertension and treatment is also reported to be associated with medication adherence [15]. Therefore, this study aimed to evaluate knowledge and acceptance associated with medication adherence among hypertensive individuals in Aceh. Acceptance level was measured using Acceptance of Illness Scale (AIS). Knowledge assessed covers individuals’ understanding of the brief pathophysiology of hypertension, pharmacological, and non-pharmacological therapy treatment as well as consequences of uncontrolled hypertension [16,17].

## 2 Methods

### 2.1 Study design

This cross-sectional study aimed to evaluate the association between knowledge and acceptance of hypertension individuals in Aceh Province, Indonesia. The investigation was conducted from March to July 2023 and ethical clearance was obtained from the Ethics Commission, Faculty of Nursing Syiah Kuala (number: 113020301221). Eligible respondents were recruited using a random sampling method, who agreed to the terms and conditions of this study by signing the informed consent form. Subsequently, the respondents were divided into three groups based on adherence level, followed by analysis of the associations with demographic characteristics, acceptance, and knowledge.

### 2.2 Sample size and randomization

Sample size was calculated using OpenEpi (https://www.openepi.com//), a web-based calculator for epidemiologic studies. The calculation was carried out using a 95 % two-sided confidence level, 80 % power, ratio of unexposed to exposed in sample of 1, and 9 % of unexposed with the outcome. Subsequently, the percentage of exposed with outcome was filled using 18 % by referring to medication adherence rate among hypertensive individuals in Aceh [4]. Although the minimum sample size required for the study was 494 respondents, a total number of 534 were recruited to increase the statistical strength. Randomization was performed by inserting the house district and number into the open-source online randomizer. The enumerator visited the house according to the order suggested by the randomizer. When one of the eligibility criteria was not met or the respondents refused to participate, the next house from the list was visited.

### 2.3 Eligibility criteria

Eligibility criteria included age >18 years old and having hypertension for at least 1 year after a doctor's diagnosis. The validity of the diagnosis was cross-checked with respondents' medical records from community health center. Hypertension was categorized based on the systolic blood pressure (SBP) including normotension (SBP<120 mmHg), pre-hypertension (SBP: 120–139 mmHg), hypertension grade I (SBP: 140–159 mmHg), hypertension grade II (SBP: 160–179 mmHg). However, individuals with secondary hypertension, emergency hypertension (≥180 mmHg), history of cerebrovascular and cardiovascular complications (such as stroke, transient ischemic attack, coronary heart diseases, and heart failure), and physical disability were excluded.

### 2.4 Survey and questionnaire

Data were collected through questionnaire distributed to randomly selected respondents with the assistance of enumerators. Age (years old), gender, education, occupation, marital status, and ethnicity were collected as demographic characteristics. Data on body height and weight were measured directly during the survey to calculate the body mass index (BMI) and nutritional status. Moreover, BMI was calculated by dividing body weight in kilograms with squared body height in meters. Respondents with BMI of <17, 18.5–25.0, 25.1–27.0, and >27 kg/m^2^ were considered underweight, normal, overweight, and obese, respectively. Medication adherence was divided into three categories, namely low (<6), moderate (6–8), and high (>8) based on the total score from Morisky Medication Adherence Scale (MMAS)-8 [18,19]. Acceptance was determined based on AIS [12], with classifications as “no” (0–18), “moderate” (19–29), and “high acceptance” (30–40). Knowledge was assessed using 22 questions adopted from a previous study [15,16], where each question could be answered with “Yes” or “No”. The themes of the questions included “blood pressure interpretation”, “factors affected blood pressure”, “lifestyle and diet modifications”, and “blood pressure-lowering therapies”.

### 2.5 Data analysis

Tabulation of the data was carried out on Microsoft Excel (Redmond, Washington, USA), while analysis was performed descriptively to obtain the frequency (*n*) of the categorical data. Meanwhile, no missing data were found in the dataset and the analytical analysis was conducted based on the ‘*X*^*2*^-test of association’, followed by ‘logistic regressions *N* outcomes’ on the program run by Jamovi version 2.3.21 (https://www.jamovi.org/). Variables with *p*-value <0.05 from the ‘*X*^*2*^-test of association’ were included in the ‘logistic regressions *N* outcomes or multinomial logistic regression. Based on the results, ‘Moderate adherence’ was selected as the model reference. A *p*-value <0.05 was considered statistically significant, while coefficients such as odd ratio (OR) and 95 % confidence interval (CI) were also calculated.

## 3 Results

### 3.1 Characteristics of respondents

Characteristics of respondents, categorized by the level of medication adherence are presented in Table 1. The number of females was more predominant than males, and the majority were between 45 and 64 years old. Both sex and age showed statistical significance at *p* < 0.05 and the distribution of respondents based on the educational level was significant (*p* < 0.001), with Senior High School graduates being the predominant group. Most respondents worked as housewives and manual workers such as fishermen, farmers, and laborers. Furthermore, there were no significant differences (*p* > 0.05) in distribution regarding marital status, occupation, ethnicity, and nutritional status.

**Table 1:** Characteristics of individuals with low, moderate, and high levels of medication adherence.

Variable	Adherence, *n* (%)			*X*^*2*^ (*p*-value)
	Low (*n* = 52)	Mod. (*n* = 330)	High (*n* = 152)	
Gender				
Males	13 (25.0)	60 (18.2)	9 (5.9)	16.2 (<0.001**)
Females	39 (75.0)	270 (81.8)	143 (94.1)	
Age (years old)				
18–44	3 (5.8)	41 (12.4)	7 (4.6)	10.5 (0.033*)
45–64	32 (61.5)	209 (63.3)	110 (72.4)	
>65	17 (32.7)	80 (24.2)	35 (23.0)	
Education				
Primary School	15 (28.8)	92 (27.9)	12 (7.9)	33.6 (<0.001**)
Junior High School	9 (17.3)	69 (20.9)	49 (32.2)	
Senior High School	20 (38.5)	118 (35.8)	67 (44.1)	
Tertiary School	5 (9.6)	35 (10.6)	22 (14.5)	
None	3 (5.8)	16 (48.5)	2 (1.3)	
Marital status				
Married	39 (75)	266 (80.6)	128 (84.2)	3.76 (0.439)
Widowed	12 (23.1)	61 (18.5)	21 (13.8)	
Unmarried	1 (1.9)	3 (0.9)	3 (1.4)	
Occupation				
Housewife	34 (65.4)	238 (72.1)	116 (76.3)	9.14 (0.166)
Manual worker	9 (17.3)	58 (17.6)	20 (13.2)	
Civil servant	3 (5.8)	23 (7.0)	10 (6.6)	
Retired	6 (11.5)	11 (3.3)	6 (3.9)	
Ethnicity				
Acehnese	47 (90.4)	306 (92.7)	135 (88.8)	2.10 (0.351)
Non-Acehnese	5 (9.6)	24 (7.3)	17 (11.2)	
Nutritional status				
Underweight	3 (5.8)	17 (5.2)	6 (3.9)	4.44 (0.617)
Normal	25 (48.1)	118 (35.8)	54 (35.6)	
Overweight	7 (13.4)	55 (16.6)	30 (19.7)	
Obese	17 (32.7)	140 (42.4)	62 (40.8)	
Acceptance				
High	3 (5.8)	39 (11.8)	95 (62.5)	154 (<0.001**)
Moderate	39 (75.0)	219 (66.4)	49 (32.2)	
Low	10 (19.2)	72 (21.8)	8 (5.3)	

Significant at *p < 0.05 or **p < 0.01.

### 3.2 Acceptance and knowledge of hypertension based on adherence level

The prevalence of high adherence to anti-hypertensive medication was 28.5 % (*n* = 534), with a significant proportion of respondents showing high acceptance, as shown in Table 1. Similarly, the majority of respondents with moderate acceptance were in the moderate adherence group, which was statistically significant (*p* < 0.001, Table 1). Data on the association between knowledge of hypertension and treatment are presented in Table 2. A significantly higher proportion of respondents with correct answers was found in the high adherence group (*p* < 0.01) for questions H6, H11, H12, H17, H18, H19, and H21. However, when respondents were asked question H12, a higher proportion answered correctly in the moderate or low adherence group (*p* = 0.006). These statistically significant proportions showed the difference in knowledge among low, moderate, and high adherence groups.

**Table 2:** The prevalence of adherence and its association with respondents’ knowledge about hypertension.

Label	Variable	Adherence, *n* (%)			*X*^*2*^ (*p*-value)
		Low (*n* = 52)	Mod. (*n* = 330)	High (*n* = 152)	
H1	High diastolic or SBP shows an increase in blood pressure.	49 (94.2)	309 (93.6)	139 (91.4)	0.893 (0.640)
H2	An increase in diastolic blood pressure shows an increase in blood pressure.	38 (73.1)	268 (81.2)	118 (77.6)	2.22 (0.329)
H3	An increase in blood pressure is caused by aging, suggesting that the treatment is not required.	50 (96.2)	318 (96.4)	144 (94.7)	0.708 (0.702)
H4	If medication intake can control blood pressure, it is not necessary to change the lifestyle.	49 (94.2)	303 (91.8)	142 (93.4)	0.632 (0.729)
H5	If individuals change their lifestyle, they no longer need treatment.	21 (40.4)	161 (48.8)	79 (52.0)	2.22 (0.330)
H6	Hypertension individuals should intake medication as they please.	13 (25.0)	93 (28.2)	101 (66.4)	68.8 (<0.001*)
H7	Hypertension medication should be taken daily.	45 (86.5)	300 (90.9)	145 (95.4)	4.85 (0.089)
H8	Hypertension individuals should take medication only when they feel unwell.	9 (17.3)	68 (20.6)	39 (25.7)	2.22 (0.329)
H9	Hypertension individuals should take a lifetime medication.	46 (88.5)	305 (92.4)	142 (93.4)	1.36 (0.507)
H10	For hypertension individuals, the best cooking method is frying.	45 (86.5)	263 (79.7)	121 (79.6)	1.40 (0.496)
H11	For hypertension individuals, the best cooking methods are boiling or grilling.	27 (51.9)	129 (39.1)	112 (73.7)	49.9 (<0.001*)
H12	Hypertension individuals can eat salty food as long as take their medication routinely.	46 (88.5)	273 (82.7)	142 (93.4)	10.3 (0.006*)
H13	Hypertension individuals should often eat fruits and vegetables.	37 (71.2)	226 (68.5)	119 (78.3)	4.92 (0.086)
H14	The type of meat for hypertension individuals is red meat.	47 (90.4)	305 (92.4)	143 (94.1)	0.876 (0.645)
H15	The type of meat for hypertension individuals is white meat.	48 (92.3)	316 (95.8)	142 (93.4)	1.84 (0.399)
H16	Hypertension individuals should not smoke a cigarette.	51 (98.1)	292 (88.5)	138 (90.8)	4.74 (0.093)
H17	Individuals with drinking habit are at risk of hypertension.	29 (55.8)	188 (57)	128 (84.2)	35.7 (<0.001*)
H18	Hypertension can cause stroke, if unmanaged.	37 (71.2)	237 (71.8)	134 (88.2)	16.3 (<0.001*)
H19	Hypertension can cause heart diseases, such as heart attack, if unmanaged.	38 (73.1)	214 (64.8)	127 (83.6)	17.8 (<0.001*)
H20	Hypertension can cause early death if untreated.	49 (94.2)	309 (93.6)	139 (91.4)	0.893 (0.640)
H21	Hypertension can cause renal failure if untreated.	36 (69.2)	205 (62.1)	74 (48.7)	10.3 (0.006*)
H22	Hypertension can cause visual impairment if untreated.	46 (88.5)	272 (82.4)	114 (75)	5.84 (0.054)

* Significant at *p* < 0.01.

### 3.3 Multinomial logistic regression

Respondents who graduated from junior *(p* = 0.002; OR = 3.84 [95%CI: 1.67–8.84]), senior (*p* = 0.001; OR = 3.94 [95%CI: 1.74–8.92]), and tertiary school (*p* = 0.018; OR = 3.56 [95%CI: 1.24–10.21]) had higher adherence. This result varied significantly compared to respondents who only graduated from primary school, as shown in Table 3. Furthermore, respondents with high-level acceptance showed a greater tendency towards high adherence compared to those with low levels (*p* < 0.001; OR = 9.14 [95%CI: 3.49–23.94]). Knowledge about dosing frequency (H6), healthy diets (H11, H12), risk of habitual alcohol intake (H17), as well as stroke and cardiovascular complications (H18, H19) correlated positively to medication adherence (*p* < 0.05). However, those with higher knowledge about renal complications showed a lower potential to have low adherence (*p* = 0.002; OR = 0.32 [95%CI: 0.16–0.66]), based on the moderate versus high adherence model.

**Table 3:** The association between demographic characteristics, knowledge, and acceptance of illness with medication adherence.

Variable	Low—Moderate		High—Moderate	
	*p*-value	OR 95 % CI	*p*-value	OR 95 % CI
Gender				
Males	Ref.		Ref.	
Females	0.450	0.76 (0.36–1.56)	0.162	1.79 (0.79–4.05)
Age (years old)				
18–44	Ref.		Ref.	
45–64	0.131	2.68 (0.75–9.63)	0.378	1.53 (0.59–3.95)
>65	0.054	3.93 (0.98–15.81)	0.215	2.00 (0.67–5.96)
Education				
Primary School	Ref.		Ref.	
Junior High School	0.727	0.85 (0.33–2.15)	0.002**	3.84 (1.67–8.84)
Senior High School	0.483	1.35 (0.58–3.11)	0.001**	3.94 (1.74–8.92)
Tertiary School	0.532	1.46 (0.44–4.84)	0.018*	3.56 (1.24–10.21)
None	0.781	1.22 (0.30–4.93)	0.654	1.47 (0.27–7.90)
Acceptance				
Low	Ref.		Ref.	
Moderate	0.481	1.34 (0.59–3.01)	0.174	1.82 (0.77–4.32)
High	0.329	0.49 (0.12–2.04)	<0.001**	9.14 (3.49–23.94)
Knowledge				
H6 (drug dose)	0.737	0.89 (0.44–1.79)	0.006**	2.08 (1.23–3.53)
H11 (cooking method)	0.061	1.83 (0.97–3.45)	0.122	1.55 (0.89–2.71)
H12 (salty foods)	0.413	1.48 (0.58–3.76)	0.061	2.20 (0.96–5.01)
H17 (drinking habit)	0.144	0.52 (0.22–1.25)	0.007**	3.03 (1.36–6.76)
H18 (stroke)	0.595	1.21 (0.60–2.45)	0.021*	2.18 (1.13–4.21)
H19 (heart attack)	0.251	1.51 (0.75–3.05)	0.727	1.12 (0.60–2.06)
H21 (renal failure)	0.129	2.01 (0.82–4.96)	0.002**	0.32 (0.16–0.66)

Significant at *p < 0.05 or **p < 0.01.

## 4 Discussion

Acceptance status was used to predict medication adherence among hypertensive individuals in Aceh. According to previous studies, individuals with high acceptance showed more likelihood of adhering to medication plan [[12], [13], [14]]. Acceptance of disease is essential for medication adherence and other health behaviors such as eating healthy foods, maintaining a positive attitude, and preventing complications [15,19,20]. In some cases, individuals might deny the diagnosis, which results in the loss of medication initiation [21]. The majority of respondents in this present study had a moderate level of acceptance, followed by high and low categories. As reported in a previous study, the prevalence of acceptance towards hypertension was relatively high compared to other chronic diseases [22].

In this study, respondents with higher education showed greater potential of following the regimen dosing prescription. A previous cross-sectional study found that individuals with at least secondary education certificates had greater medication adherence compared to those with primary or no education [23]. In Panama, males who only attended primary school tended to possess non-adherence to medication [21], including those lacking awareness about the risk of developing complications [24]. Moreover, the lack of awareness about hypertension is not only limited to adult but also among the younger population [25].

Respondents possessing knowledge about the importance of appropriate dosing frequency, low fat, and sodium diets, as well as healthy habits (not drinking alcohol) showed a higher tendency towards high-level adherence. Similarly, those with knowledge about the risk of developing stroke and cardiovascular complications were found to have better adherence. This shows that sufficient knowledge about hypertension can increase the motivation of individuals to perform better self-care routines, including long-term medication adherence [15]. In a published review, adherence level of individuals living in low-to-middle-income countries was determined by drinking behavior [26].

This study showed that individuals knowledgeable about renal failure as a complication of uncontrolled hypertension tended to have lower medication adherence plans. This may be related to a widely believed myth that anti-hypertensive medication could negatively impact kidneys [27]. Furthermore, fear of side effects has been recognized in a previous study as a common barrier to medication adherence among the adult population with hypertension [28].

Based on the results, interventions aiming at improving adherence to anti-hypertensive medication among Acehnese population are recommended to promote knowledge about hypertension complications and prevention. Educational materials for these interventions must show the importance of healthy diets and habits to control blood pressure. A previous study conducted by a series of health promotion programs in Kenyan rural areas has reported an increase in knowledge about hypertension [29], showing the importance of adjusting to local culture in educating the community. Similarly, a culture-based educational program was reported to increase medication adherence, leading to a decrease in the average blood pressure [30].

Despite the valuable information from this study, the limitations included failure to observe the comorbidities among respondents and perception of the treatment, thereby influencing adherence level. The presence of comorbidities often requiring individuals to receive polypharmacy treatment was identified as a significant risk factor for low medication adherence [31]. In another study, polypharmacy treatment and multiple doses per day reduce medication adherence [32], while suggesting that early use of fixed-dose single-pill combinations of antihypertensive drugs could improve adherence [32]. Therefore, future studies should collect data on the presence of comorbidities, polypharmacy treatment, and multiple doses daily. Perceived risk and efficacy of the treatment were suggested as adherence determinants, specifically in young and adult populations [26]. Since the respondents were only recruited from Aceh Province, the results had limited generalizability to the Indonesian population. Medication adherence was also measured using a self-report questionnaire which could be influenced by recall bias. As a cross-sectional study, the bias from confounding and residual confounding factors were not controlled, specifically with the proportional number of subjects according to age and gender, limiting results interpretation. Consequently, future studies are recommended to use better design by applying real-time monitoring data or metabolomic screening [33,34] as alternatives to self-reported surveys.

## 5 Conclusions

In conclusion, this study showed that the level of acceptance correlated with adherence of hypertension individuals in following the pharmacological treatment plan. The results showed that respondents with higher educational backgrounds had a greater tendency towards a high level of adherence. The major knowledge areas that contributed to increasing adherence included dosing frequency, low-fat and sodium diets, as well as the effect of drinking alcohol. Based on the results, knowledge about the consequences of uncontrolled hypertension such as stroke and cardiovascular complications was identified as the most significant contributor to higher adherence. Meanwhile, individuals with knowledge about renal complications showed low adherence to anti-hypertensive medication due to the fear of side effects. These results could be used as the basis for developing educational materials to improve medication adherence in Aceh, Indonesia.

## Ethical clearance

This study was reviewed and approved by the Ethics Commission, Faculty of Nursing Syiah Kuala, with the approval number: 113020301221. All participants/patients (or their proxies/legal guardians) provided informed consent to participate in the study.

## Funding

This study was supported by the Ministry of Education, Research and Technology (062/E5/PG.02.00.PL/2023)

## Ethics declarations

• This study was reviewed and approved by an ethic committee at Universitas Syiah Kuala, with the approval number: 11302030122.
• All participants/patients (or their proxies/legal guardians) provided informed consent to participate in the study.

## CRediT authorship contribution statement

**Sri Andala:** Writing – review & editing, Writing – original draft, Visualization, Software, Methodology, Investigation, Data curation, Conceptualization. **Hizir Sofyan:** Writing – review & editing, Writing – original draft, Validation, Supervision, Resources, Methodology, Funding acquisition, Data curation, Conceptualization. **Kartini Hasballah:** Writing – review & editing, Writing – original draft, Validation, Resources, Project administration, Methodology, Data curation, Conceptualization. **Marthoenis:** Writing – review & editing, Writing – original draft, Validation, Software, Methodology, Formal analysis, Data curation, Conceptualization, Writing – review & editing, Writing – original draft, Validation, Software, Methodology, Formal analysis, Data curation, Conceptualization.

## Declaration of competing interest

The authors declare that they have no known competing financial interests or personal relationships that could have appeared to influence the work reported in this paper.
